# Modified Tibial Tuberosity Advancement Rapid in a Dog with One Contralateral Amputated Limb

**DOI:** 10.3390/vetsci9090476

**Published:** 2022-09-02

**Authors:** Ciprian Ober, Mădălina Dragomir, Andreea Aștilean, William McCartney, Christos Yiapanis, Joshua Milgram

**Affiliations:** 1 Department of Surgery, Faculty of Veterinary Medicine, University of Agricultural Sciences and Veterinary Medicine, 400372 Cluj-Napoca, Romania; 2 North Dublin Orthopaedic Animal Hospital, D13 K5H0 Dublin, Ireland; 3 Cyvets Veterinary Centre, Paphos 8025, Cyprus; 4 Department of Small Animal Surgery, Koret School of Veterinary Medicine, Rehovot 76100, Israel

**Keywords:** amputation, modified TTA rapid, stifle joint, cranial cruciate ligament disease, dog

## Abstract

**Simple Summary:**

Cranial cruciate ligament disease is a very common source of pelvic limb lameness in dogs, and many techniques have been used to resolve this condition. Tibial tuberosity advancement (TTA) rapid technique is a new simplified option with very good results reported. Adding a small Steinmann pin distal to the cage prevents avulsion of the tibial tuberosity by quadriceps mechanism. In this report, we describe the first modified TTA rapid technique in a dog with a contralateral amputated limb. The dog was a five-year-old mixed breed with amputated right pelvic limb and difficulty walking. The dog started to walk unassisted second day postoperative, and at three months follow-up evaluation it showed no lameness and the osteotomy was completely healed.

**Abstract:**

Cranial cruciate ligament disease (CCLD) is one of the most frequent causes of hindlimb lameness in dogs. Tibial tuberosity advancement (TTA) is a common surgery performed for CCLD. A modified, simplified technique (TTA Rapid) is also reported to have very good clinical outcomes. In this paper, we report a modified TTA Rapid technique to treat a CCLD in a dog with an amputated contralateral hindlimb. A 5-year-old mixed breed dog presented with amputated right hindlimb and difficulty walking. Pain and positive drawer sign were present at manipulation of left stifle joint. Radiographic findings of the stifle joint confirmed the presence of moderate osteoarthritis associated with CCLD, and modified TTA Rapid procedure was performed. Recovery from surgery was uneventful, and the dog was able to stand by his own by the second day postoperative. At three months follow-up evaluation, the dog was free of lameness and the osteotomy site was completely healed. This paper describes the first modified TTA rapid osteotomy technique performed in a dog with a contralateral amputated hindlimb.

## 1. Introduction

Cranial cruciate ligament disease is one of the most common causes of hindlimb lameness and stifle osteoarthritis in dogs [1,2]. Surgical techniques to restabilize the joint, with either static or dynamic repairs, are performed to neutralize the tibiofemoral shear forces in a CrCL-deficient knee [3]. A popular surgical technique used to achieve stifle joint stability that neutralizes the tibiofemoral shear forces dynamically in a CrCL-deficient stifle is tibial tuberosity advancement (TTA) [4,5,6]. TTA has been reported to functionally stabilize the stifle joint during weight bearing by neutralizing the cranial tibiofemoral shear force by advancing the tibial tuberosity [3]. This is accomplished by an osteotomy of the tuberosity in the frontal plane with advancement of this bone fragment [4]. Tibial tuberosity advancement has proven to be a technique with clinical results similar to those of other osteotomy techniques [3,7,8]. Technique modifications have eliminated the plate, instead relying only on the cage to provide the advancement and stability [9]. Modified techniques include the Modified Maquet Procedure [9], TTA Rapid [10], and TTA-2 [11,12]. These techniques led to the development of alternative cage designs and techniques to orient the tibial osteotomy at a fixed distance along the cranial tibial shaft [13]. The osteotomy length corresponds to the degree of tuberosity advancement (i.e., cage width) desired. The mechanics of different TTA models have also been validated in one experimental model [14]. In our clinical case, the modified TTA rapid technique refers to the insertion of the Steinmann pin distal to the cage to prevent tibial tuberosity avulsion or fracture.

According to the best of our knowledge, this report is the first modified TTA Rapid performed in a dog with cranial cruciate ligament deficient stifle with amputated contralateral hindlimb. 

## 2. Detailed Case Description

**Figure 1:**
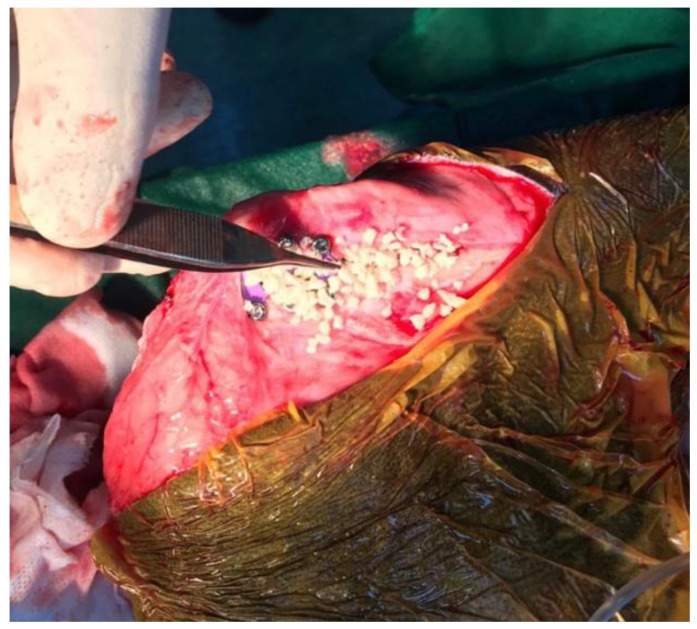
Tricalcium phosphate granules as osteoconductive material used to fill the spaces in the osteotomy gap.

A 5-year-old, 38-kg mixed breed castrated male dog (BCS 4 of 9) was presented because of progressive lameness of left hindlimb. The contralateral limb was amputated two years prior to referral because of severe injuries after a road traffic accident. The dog was able to walk a few steps, but recumbency position was preferred most of the time at presentation. Positive cranial drawer sign was observed, which is an indication of CCLD. Mediolateral and craniocaudal radiographs of the left stifle were performed using sedation (acepromazine, 0.01 mg/kg IV and dexmedetomidine, 4 mg/kg IV). Tibial axis method was used to determine the cage size for optimal advancement. Acepromazine (0.01 mg/kg IV) and methadone (0.2 mg/kg IV) were used for premedication. Cephazolin (22 mg/kg IV) was administered 30 min before induction of anesthesia. Induction of anesthesia was performed with propofol (3 mg/kg IV) and maintained with isoflurane (1000 mg/g). Bupivacaine 0.5% (1 mg/kg) was administered epidural in the lumbosacral junction. Carprofen (2 mg/kg IV) was also administered. Dorsal recumbency was the first position, and the affected hindlimb was aseptically prepared for surgery. Remnants of the torn CCL were removed via a medial miniarthrotomy. Inspection of the medial and lateral meniscus was performed with a meniscal probe, and no meniscal tears were observed. The joint was irrigated with sterile saline solution. The joint capsule was closed with 4-0 absorbable monofilament suture in a simple continuous pattern. The dog was repositioned in lateral recumbency, with the operated limb downwards, parallel with the ground. The previous skin incision was enlarged distally to the tibial crest in order to perform the TTA rapid procedure. A 1.6-mm Steinmann pin was placed through the joint capsule just cranial to Gerdi’s tubercle for the TTA’s osteotomy guidance. This was done perpendicular to the sagittal plane. The TTA rapid spreader was used to advance the tibial crest and insert the 12-mm cage. Self-tapping 2.4-mm titanium cortical screws were used to fix the cage in place. Hemorrhage of one nutrient artery was observed before inserting a caudal screw, but it resolved at screw insertion. The spaces in the osteotomy gap and the cage were filled with tricalcium phosphate granules (Syntoss, DSI Israel) (Figure 1). 

**Figure 2:**
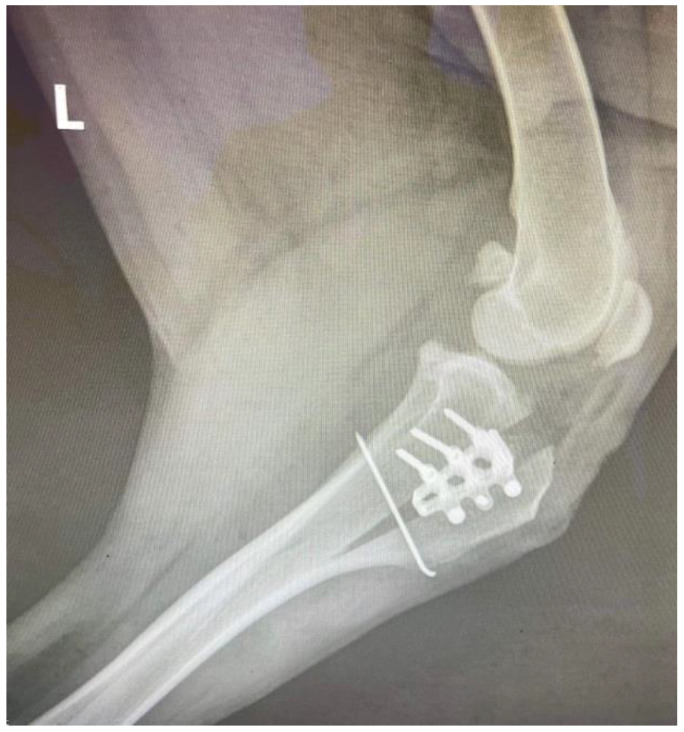
Immediate postoperative appearance of the implants.

A-1.6 mm Steinmann pin was inserted after cage fixation in order to prevent tibial crest avulsion or fracture and implant failure. Mediolateral postoperative radiographs were necessary to evaluate the cage and screws’ positions (Figure 2). 

**Figure 3:**
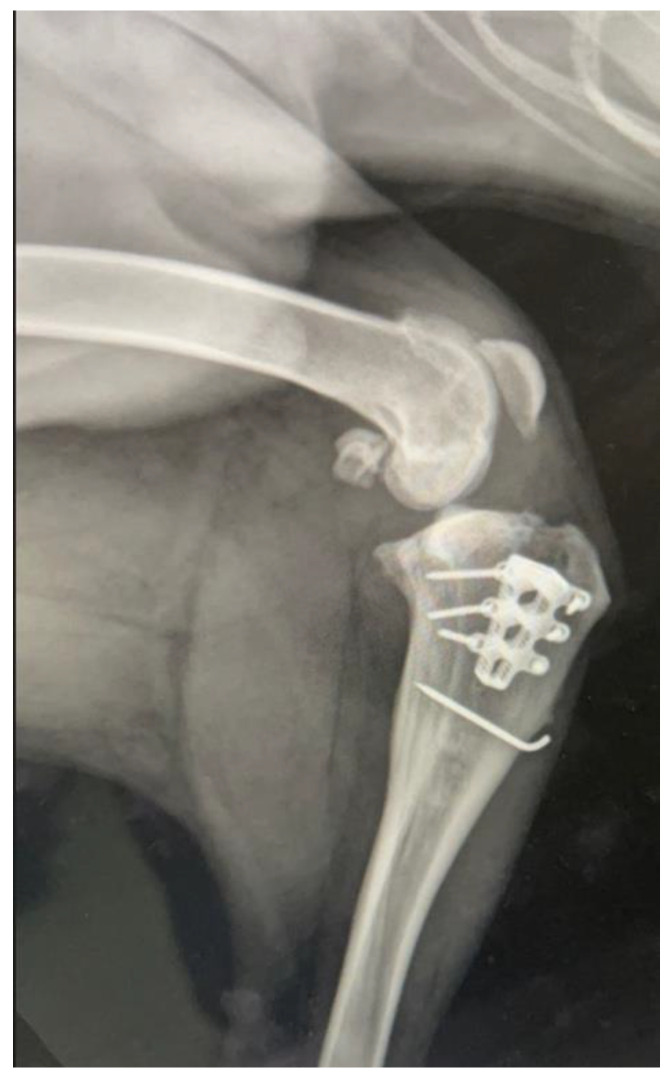
Complete bone healing 3 months postoperative.

The dog was administered oral firocoxib (5 mg/kg once daily) for 10 days and amoxicillin plus clavulanic acid for 10 days. Restriction of activities was recommended for 7 weeks. Lameness evaluation was performed two times per month, and final radiographic assessment was performed at 3 months postoperatively to evaluate implants and the healing of the osteotomy. The healing was defined as the presence of bridging callus formation around and inside the cage. Final healing was defined when trabecular bone was present in the mineralized callus [15]. A slight thickening of the patellar ligament was observed on the follow-up ultrasonography at 2 months postoperatively but resolved at three months. Clinical follow-up was assessed two times per month, and final radiographic follow-up was recorded at 3 months postoperatively. The dog had complete bone healing at 3 months (Figure 3). 

**Figure 4:**
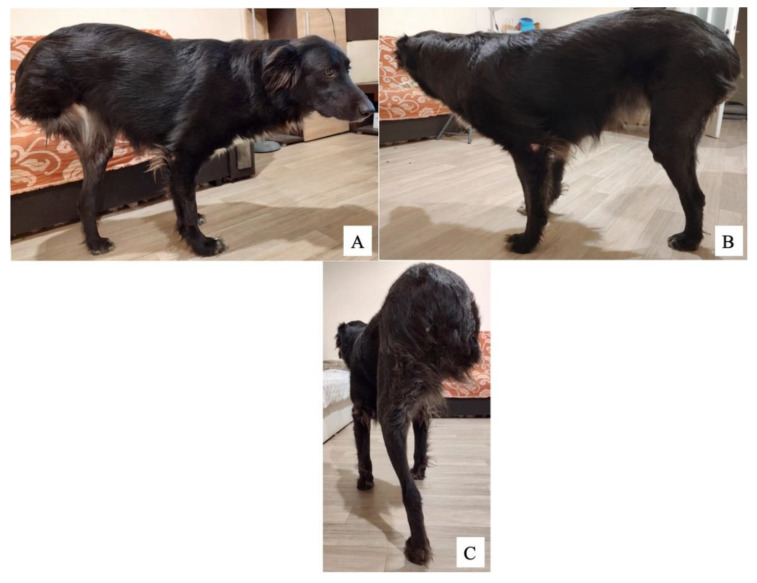
Photos of dog at 3 months postoperative: left lateral (**A**), right lateral (**B**), and caudal (**C**). Note the alopecic operated area on the left medial tibial crest (**A**).

Three months postoperatively, the dog had an excellent outcome, with the ability to walk around freely (Figure 4). The owner was very satisfied with the outcome and reported a return to pre-injury.

## 3. Discussion

This paper reports a modified TTA rapid technique and immediate postoperative outcome in a dog with an amputated contralateral limb. TTA rapid represents a well-known and effective technique for the treatment of CCL disease [16,17], and we use this technique as the standard of care in dogs with cranial cruciate ligament insufficiency. The aim of the TTA rapid is to maintain the clinical success of the TTA technique, using fewer implants. The distal cortex of the tibial tuberosity will be maintained, annihilating the pull of the quadriceps muscle group. However, tibial crest avulsion or fracture may still appear in some cases [10]. The reported technique in this paper is a modification of the conventional TTA rapid technique, where an attempt is made to prevent avulsion or fracture of the tibial crest, and subsequent implant failure, due to the pull of the quadriceps muscle. The modified technique includes the insertion of a 1.6-mm Steinmann pin during the surgery immediately after cage placement. It was previously demonstrated that fixating the osteotomy with tension band wiring increases the strength of the fixation and decreases the risk of implant failure [14]. No complication was observed at the last radiographic assessment three months postoperative. 

A preplaced drill hole (Maquet hole) was proposed at the termination site of the osteotomy to prevent fissure or propagation of the osteotomy past this predetermined location [15,18,19]. The risk of fracture of the distal tibial tuberosity, or even the tibia, from propagation of the osteotomy has been described in 20% of procedures [15]. Little is known about the diameter of a Maquet hole depending on the cage size. The true requirement of a Maquet hole is also debatable [19]. We decided not to perform the procedure for a Maquet hole in our patient.

In the classic TTA technique, in the bone gap that resulted between the tibial crest and the tibia, an autograft, allograft, xenograft, or a bone substitute were used [4,20]. There was no observed need for bone grafting to achieve good radiographic healing in a previous study [21], but we used tricalcium phosphate granules to enhance bone healing. 

Patellar desmopathy has been reported as a minor complication both in tibial plateau levelling osteotomy (TPLO) [22] and TTA [23]. The clinical significance of patellar tendon desmopathy is not known, and specific treatment of this condition was not required. We have noticed this aspect in our patient at two months postoperatively. Interestingly, at the last ultrasonographic assessment the condition was not present anymore.

Possible disadvantages of this modified technique, such as tibial crest avulsion or fracture, as well as implant migration and infection, need to be considered. The follow-up time for our patient was short, and this is a limitation of the study. Future studies will have to demonstrate if a Maquet hole is needed to prevent the propagation of the osteotomy line and fissures of the cortical bone. Prospective clinical studies are necessary to demonstrate the role, the adequate size, of the pin and potential complications.

## 4. Conclusions

The presented study described a modified TTA rapid technique in a dog with a contralateral amputated hindlimb. The report showed that this method may be a viable alternative for treating CCL disease in dogs with amputated contralateral limbs. These findings show the importance of recognizing such a condition in the process of decision-making for a favorable outcome.

Future studies are necessary to establish if the results are the same in larger populations in the long term. We assume that this would be a difficult task, as it is not easy to find a population of dogs with CCL disease and an amputated contralateral limb.

## Data Availability

Not applicable.
